# Evaluation of the influenza sentinel surveillance system in Madagascar, 2009–2014

**DOI:** 10.2471/BLT.16.171280

**Published:** 2017-02-21

**Authors:** Alain Rakotoarisoa, Laurence Randrianasolo, Stefano Tempia, Julia Guillebaud, Norosoa Razanajatovo, Lea Randriamampionona, Patrice Piola, Ariane Halm, Jean-Michel Heraud

**Affiliations:** a Direction de la Veille Sanitaire et de la Surveillance Epidémiologique, Ministry of Public Health, Antananarivo, Madagascar.; b Epidemiology Unit, Institut Pasteur de Madagascar, Antananarivo, Madagascar.; c Influenza Division, Centers for Disease Control and Prevention, Atlanta, United States of America.; d National Influenza Centre, Virology Unit, Institut Pasteur de Madagascar, Ambatofotsikely, BP 1274, Antananarivo, Madagascar.; e Epidemiology and Surveillance Unit, Indian Ocean Commission, Ebène, Mauritius.

## Abstract

**Problem:**

Evaluation of influenza surveillance systems is poor, especially in Africa.

**Approach:**

In 2007, the Institut Pasteur de Madagascar and the Malagasy Ministry of Public Health implemented a countrywide system for the prospective syndromic and virological surveillance of influenza-like illnesses. In assessing this system’s performance, we identified gaps and ways to promote the best use of resources. We investigated acceptability, data quality, flexibility, representativeness, simplicity, stability, timeliness and usefulness and developed qualitative and/or quantitative indicators for each of these attributes.

**Local setting:**

Until 2007, the influenza surveillance system in Madagascar was only operational in Antananarivo and the observations made could not be extrapolated to the entire country.

**Relevant changes:**

By 2014, the system covered 34 sentinel sites across the country. At 12 sites, nasopharyngeal and/or oropharyngeal samples were collected and tested for influenza virus. Between 2009 and 2014, 177 718 fever cases were detected, 25 809 (14.5%) of these fever cases were classified as cases of influenza-like illness. Of the 9192 samples from patients with influenza-like illness that were tested for influenza viruses, 3573 (38.9%) tested positive. Data quality for all evaluated indicators was categorized as above 90% and the system also appeared to be strong in terms of its acceptability, simplicity and stability. However, sample collection needed improvement.

**Lessons learnt:**

The influenza surveillance system in Madagascar performed well and provided reliable and timely data for public health interventions. Given its flexibility and overall moderate cost, this system may become a useful platform for syndromic and laboratory-based surveillance in other low-resource settings.

## Résumé

**^Problème^:**

^ L'évaluation des systèmes de surveillance de la grippe est médiocre, en particulier en Afrique.^

**^Approche^:**

^ En 2007, l'Institut Pasteur de Madagascar et le ministère malgache de la Santé publique ont mis en œuvre un système national de surveillance prospective, syndromique et virologique, des syndromes grippaux. En évaluant les performances de ce système, nous avons repéré certaines lacunes ainsi que des moyens d'améliorer l'utilisation des ressources. Nous avons examiné l'acceptabilité, la qualité des données, la flexibilité, la représentativité, la simplicité, la stabilité, l'actualisation et l'utilité de ce système, et avons développé des indicateurs qualitatifs et/ou quantitatifs pour chacun de ces aspects.^

**^Environnement local ^:**

^Jusqu'en 2007, le système de surveillance de la grippe à Madagascar n'était opérationnel qu'à Antananarivo et les observations qui étaient faites ne pouvaient pas être extrapolées au pays entier.^

**^Changements significatifs^:**

^ En 2014, le système était utilisé sur 34 sites sentinelles, sur l'ensemble du pays. Des prélèvements nasopharyngés et/ou oropharyngés ont été effectués sur 12 sites, avant d'être soumis à un test pour rechercher le virus de la grippe. Entre 2009 et 2014, 177 718 cas de fièvre ont été détectés, sur lesquels 25 809 (14,5%) ont été classés comme syndromes grippaux. Sur les 9192 prélèvements, effectués sur des patients qui présentaient des syndromes grippaux, sur lesquels on a recherché des virus de la grippe, 3573 (38,9%) se sont révélés positifs. La qualité des données, pour tous les indicateurs évalués, a été classée au-dessus de 90% et le système présentait également de bons résultats au niveau de son acceptabilité, de sa simplicité et de sa stabilité. Cependant, la réalisation de prélèvements devait être améliorée.^

**^Leçons tirées^:**

^ Le système de surveillance de la grippe à Madagascar présentait de bons résultats et fournissait des données fiables et actualisées pour les interventions de santé publique. Compte tenu de sa flexibilité et de son coût relativement modéré, ce système pourrait devenir une plate-forme utile pour la surveillance syndromique et en laboratoire dans d'autres pays à faibles ressources.^

## Resumen

**Situación:**

La evaluación de los sistemas de vigilancia de la gripe es escasa, sobre todo en África.

**Enfoque:**

En 2007, el Instituto Pasteur de Madagascar y el Ministerio de Salud Pública de Madagascar implementaron un sistema nacional para la futura vigilancia sindrómica y epidemiológica de enfermedades similares a la gripe. Al evaluar el rendimiento de este sistema, se identificaron lagunas y formas de fomentar el mejor uso de los recursos. Se investigaron la aceptación, la calidad de la información, la flexibilidad, la representación, la simplicidad, la estabilidad, el momento y la utilidad, y se desarrollaron indicadores cualitativos y/o cuantitativos para cada uno de estos atributos.

**Marco regional:**

Hasta 2007, el sistema de vigilancia de la gripe en Madagascar operaba únicamente en Antananarivo, y las observaciones realizadas no podían extrapolarse al resto del país.

**Cambios importantes:**

En 2014, el sistema abarcaba 34 sitios centinela en todo el país. En 12 sitios, se recogieron muestras nasofaríngeas y/o bucofaríngeas, que se sometieron a pruebas del virus de la gripe. Entre 2009 y 2014 se detectaron 177 718 casos de fiebre, 25 809 (14,5%) de los cuales se clasificaron como casos de enfermedades similares a la gripe. De las 9 192 muestras de pacientes con enfermedades similares a la gripe sometidos a pruebas del virus de la gripe, 3 573 (38,9%) resultaron positivas. La calidad de los datos para todos los indicadores evaluados se categorizó como superior al 90% y el sistema también parecía ser sólido en cuanto a su aceptación, simplicidad y estabilidad. No obstante, la recogida de muestras necesitaba mejorar.

**Lecciones aprendidas:**

El sistema de vigilancia de la gripe en Madagascar obtuvo buenos resultados y ofreció información fiable y oportuna para las intervenciones de salud pública. Dada su flexibilidad y el coste moderado general, este sistema podría convertirse en una plataforma útil para la vigilancia sindrómica y en laboratorios en otros entornos con pocos recursos.

## ملخص

**المشكلة:**

ضعف تقييم نظم ترصد الإنفلونزا خاصةً في أفريقيا.

**الأسلوب:**

نفذ كلاً من *معهد باستور * ووزارة الصحة العامة في مدغشقر نظامًا شاملاً على مستوى البلاد لرصد الحالات المحتملة لانتشار المتلازمات المَرضية والفيروسية للأمراض المماثلة للإنفلونزا وذلك في عام 2007. وبتقييم أداء هذا النظام، حددنا ثغرات وطرقًا لتعزيز أفضل استخدام للموارد. وقد تحرينا المقبولية، وجودة البيانات، والمرونة، وتمثيل العينات، والبساطة، والاستقرار، والتوقيت، والفائدة، كما وضعنا المؤشرات النوعية و/أو الكمية لكل سمة من هذه السمات.

**المواقع المحلية:**

حتى عام 2007، تم تشغيل نظام رصد الأنفلونزا في مدغشقر في أنتاناناريفو فقط، وتعذر استقراء الملاحظات للبلد بأكمله

**التغييرات ذات الصلة:**

بحلول عام 2014، غطى النظام 34 موقعًا رصديًا في جميع أنحاء البلاد. وتم جمع عينات بلعومية و/أو فموية بلعومية واختبارها لاكتشاف فيروس الإنفلونزا في 12 موقعًا. تم اكتشاف 177,718 حالة حمى في الفترة ما بين عامي 2009 و2014، وتم تصنيف 25,809 (‏14.5‏%) حالة من هذه الحالات على أنها أمراض مماثلة للإنفلونزا. وكان من بين 9192 عينة من المرضى المصابين بأمراض مماثلة للإنفلونزا والتي تم اختبارها لاكتشاف فيروس الإنفلونزا، ثبتت إصابة 3573 (‏38.9‏%) حالة. أشار تصنيف جودة البيانات لجميع المؤشرات التي تم تقييمها إلى نسبة تتعدى 90‏‏%، كما ظهر النظام قويًا فيما يتعلق بالمقبولية والبساطة والاستقرار. ومع ذلك، يلزم إدخال التحسين على عملية جمع العينات.

**الدروس المستفادة:**

حقق نظام رصد الإنفلونزا في مدغشقر أداءً جيدًا وقدّم بيانات موثوقة وفي الوقت المناسب لإجراء تدخلات الصحة العامة. قد يكون هذا النظام منصة مفيدة لرصد المتلازمات المرضية وعمليات الرصد في المختبرات في المواقع الأخرى قليلة الموارد وذلك بسبب مرونة هذا النظام وتكاليفه المعقولة بشكل عام.

## 摘要

**问题:**

对流感监测系统的评估不足，尤其是在非洲。

**方法:**

2007 年，*马达加斯加巴斯德研究所 (Institut Pasteur de Madagascar)* 和马达加斯加公共卫生部启用了一个全国性系统，对流感样疾病进行前瞻性综合征监测和病毒学监测。 在评估该系统绩效的过程中，我们发现了不足之处以及促进资源最佳利用的方式。 我们研究了可接受性、数据质量、灵活性、代表性、简单性、稳定性、及时性和有用性，并且为以上各属性制定了定性和/或定量指标。

**当地状况:**

在 2007 年以前，马达加斯加的流感监测系统仅在塔那那利佛运行，并且观察结果无法外推到整个国家。

**相关变化:**

截止 2014 年，该系统覆盖全国 34 个哨点。 我们在 12 个哨点采集了鼻咽和/或口咽样本并进行了流感病毒检测。 在 2009 年至 2014 年期间，我们发现了 177 718 宗发热病例，其中 25 809 (14.5%) 宗被归类为流感样疾病病例。 在进行流感病毒检测的 9192 个流感样疾病患者的样本中，3573 (38.9%) 个样本的检测结果呈阳性。 所有评估指标下的数据质量均超过 90%，并且系统在其可接受性、简单性和稳定性方面似乎也非常卓越。 然而，样本采集需要改进。

**经验教训:**

马达加斯加流感监测系统运行情况良好，并且为公共卫生干预提供可靠、及时的数据。 鉴于其灵活性和总体适中的成本，该系统可能会成为其他资源匮乏的地区进行综合征监测和实验室监测的有用平台。****

## Резюме

**Проблема:**

Неудовлетворительная оценка систем эпиднадзора за гриппом, особенно в Африке.

**Подход:**

В 2007 году Институтом Пастера в Мадагаскаре (*Institut Pasteur de Madagascar*) и Министерством здравоохранения Мадагаскара была внедрена национальная система для перспективного синдромного и вирусологического наблюдения гриппоподобных заболеваний. При проведении оценки эффективности этой системы авторы определили недостатки и способы стимулирования наиболее эффективного использования ресурсов. Авторы исследовали приемлемость, качество данных, гибкость, репрезентативность, простоту, стабильность, своевременность и полезность и разработали качественные и/или количественные показатели для каждой из этих характеристик.

**Местные условия:**

До 2007 года система эпиднадзора за гриппом на Мадагаскаре действовала только в Антананариву и полученные результаты наблюдений было невозможно экстраполировать на всю страну.

**Осуществленные перемены:**

К 2014 году система охватывала 34 поста наблюдения по всей стране. На 12 постах были отобраны и протестированы на наличие вируса гриппа мазки из носоглотки и/или ротоглотки. В период с 2009 по 2014 год было выявлено 177 718 случаев лихорадки, 25 809 (14,5%) из этих случаев были классифицированы как случаи гриппоподобных заболеваний. Из 9192 проб, взятых у пациентов с гриппоподобными заболеваниями и протестированных на наличие вирусов гриппа, 3573 (38,9%) дали положительный результат. Качество данных для всех оцениваемых показателей было классифицировано как превышающее 90%. Система продемонстрировала хорошие показатели с точки зрения своей приемлемости, простоты и стабильности. Тем не менее отбор проб нуждается в улучшении.

**Выводы:**

Система эпиднадзора за гриппом в Мадагаскаре хорошо зарекомендовала себя и позволяла получать надежные и своевременные данные для мероприятий в области общественного здравоохранения. С учетом гибкости и умеренной стоимости этой системы она может стать полезной платформой для синдромного и лабораторного наблюдения в условиях ограниченности ресурсов.

## Introduction

The World Health Organization (WHO) recommends that, from no more than two years after implementation, influenza surveillance systems should be periodically and comprehensively evaluated.^1^ Such evaluations may enable shortfalls to be identified, performance to be improved and data reliability to be assessed. Although several influenza surveillance systems have been established in Africa,^2^^,^^3^ data on the performance of influenza surveillance in Africa are scarce.

## Local setting

Madagascar is a low-income country with a health system that faces numerous challenges – including problems in the timely detection of disease outbreaks and the mounting of effective responses to such outbreaks. Although there has been an influenza surveillance system in Madagascar since 1972, in 2007 this system covered only six primary health centres – all located in the capital city of Antananarivo. Between 2002 and 2006, each of the six health centres collected up to five specimens weekly from patients presenting with influenza-like illness (ILI). Staff from the national influenza centre in Antananarivo collected these specimens twice a week. Only one centre reported weekly aggregated data on the numbers of ILI cases recorded among all consultations. The pre-2007 system could monitor influenza activity only in the capital city. Thus, for influenza pandemic preparedness and to satisfy the 2005 International Health Regulations,^4^ it became important to implement influenza surveillance throughout Madagascar.

## Approach

In 2007, in collaboration with the Malagasy Ministry of Public Health, the Institut Pasteur de Madagascar initiated a countrywide system for the prospective syndromic and virological surveillance of fever.^3^^,^^5^ The system was designed to enable the daily collection of data on ILI, the daily reporting of the data to staff at the Institut Pasteur de Madagascar – via a short message service-based system – and the collection of samples to be tested for influenza virus. The main aim of the syndromic surveillance, which was integrated in the general practice of the clinicians at the sentinel sites, was the prompt detection of any influenza-related unusual event, outbreak or seasonal epidemic, especially in areas where laboratory-confirmed diagnoses were difficult to obtain.

**Fig. 1:**
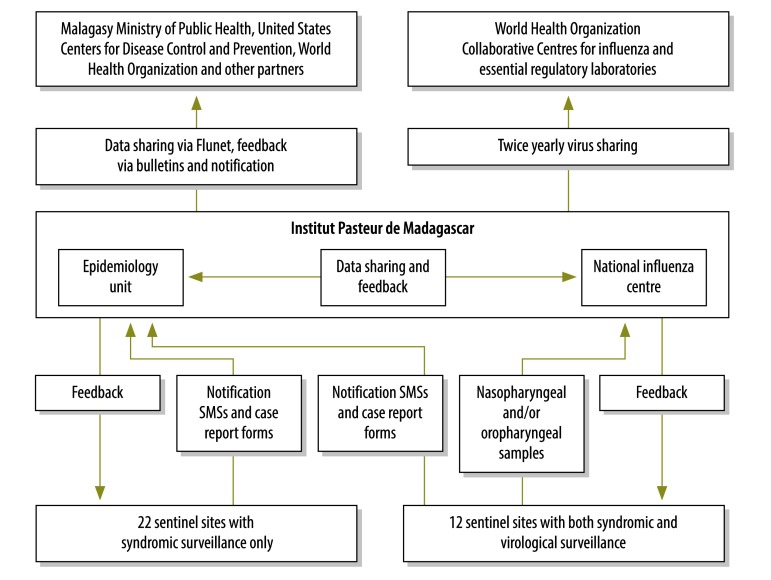
Flowchart showing the implementation of the national system for the surveillance of influenza-like and other febrile illnesses, Madagascar, 2009–2014

To check that the reliable data needed for effective public health interventions were being generated, we evaluated the influenza surveillance component of the fever surveillance system between January 2009 and December 2014. During the study period, influenza surveillance – nested within the fever surveillance – was implemented in 34 public or private health-care facilities spread across Madagascar (available from the corresponding author). Each day, trained staff at each of these sentinel sites were supposed to report, via text messages to the Institut Pasteur de Madagascar, the age-stratified numbers of outpatients who had presented with fever, i.e. a temperature of  at least 38 °C (Fig. 1). For each person with fever that gave verbal informed consent, a standardized paper-based case report form should have been used to record demographic characteristics, clinical symptoms and date of illness onset. Case report forms should have been sent to the Institut Pasteur de Madagascar weekly, by express courier. All the data sent were entered into a central electronic database. If incomplete or inconsistent data were detected, queries were sent to the corresponding sentinel sites. Each day, a time–trend analysis of the syndromic surveillance data was implemented so that any peaks in ILI incidence – above a pre-established threshold – could be detected rapidly. Clinicians at the sentinel sites identified cases of ILI, among the fever cases, using standard WHO case definitions.^6^^,^^7^ Daily, weekly and monthly reports were generated at the Institut Pasteur de Madagascar and shared with the sentinel sites and other key stakeholders.

SMS: short message service.

Weekly, at 12 of the sentinel sites, nasopharyngeal and/or oropharyngeal samples were collected from up to five patients with ILI and shipped to the national influenza centre for influenza testing, as previously described.^8^^,^^9^

No financial incentives were provided to the health centre staff for their surveillance-related activities but medical equipment, stationery and training were provided to support such activities.

To evaluate the influenza surveillance system, we followed the relevant guidelines of the United States Centers for Disease Control and Prevention^10^^,^^11^ and considered eight key attributes. For each attribute, specific quantitative and/or qualitative indicators were developed and scored (Table 1).

**Table 1:** Key findings from the evaluation of the influenza surveillance system in Madagascar, 2009–2014

Attribute, issue Data quality	Indicator	Key findings	Score^a^
Does the information submitted contain all mandatory and/or requested data items and are the data recorded valid?	Proportion of expected SMS messages and CRF that were received	93.0% (IQR:70.2–98.1) of expected SMS and 89.5% (IQR: 40.9–95.3) of expected CRF	3
	Proportion of SMS and CRF without missing or inconsistent value for selected key variables^b^	99.9% (44 203/44 252) of SMS and 96.6% (117 397/121 543) of CRF	3
	Proportion of ILI cases that met the case definition	94.9% (24 490/25 809)	3
	Proportion of sampled ILI cases that met the sampling criteria^c^	99.5% (9251/9293)	3
	Proportion of sampled ILI cases with available laboratory results	98.9% (9192/9293)	3
	Proportion of collected variables included in the WHO recommended minimum data collection for influenza sentinel surveillance	Data on antiviral treatment and underlying medical conditions were not collected	2
**Timeliness**			
Are the data and samples from the surveillance system collected and dispatched without delay?	Proportion of SMS texts sent within 48 hours of reference day	69.8% (IQR: 59.8–77.1)	2
	Proportion of data collection forms received by IPM within 7 days of data collection	90.3% (IQR: 81.2–98.1)	3
	Proportion of samples received by IPM within 48 hours of collection	45.9% (IQR: 29.9–72.7)	1
	Proportion of weekly reports issued within the target date	90.1% (281/312)	3
**Representativeness**			
Are the data collected on influenza by the surveillance system representative of the general population in Madagascar?	Geographical coverage	Surveillance sites located in all provinces	3
	Inclusion of all age groups	Although all age groups were eligible, median age was only 4 years (range: 1 day–91 years)	2
**Simplicity**			
Do the surveillance staff find the system easy to implement?	Surveillance staff’s perceptions of how easy certain surveillance activities are to use – categorized as very difficult, difficult or easy	Of 50 respondents, the collection of aggregated data, the completion of CRF and SMS-based data transfer were reported to be easy by 47, 50 and 50, respectively	3
	Performance of the courier in collecting CRF from sentinel sites	Of 50 respondents, 27 reported that they had rarely or never experienced delays in the collection of CRF	1
	Performance of the courier in collecting samples from sentinel sites	All the 18 respondents from sites where samples were collected reported that they had rarely or never experienced delays in the collection of samples	3
**Acceptability**			
Do the surveillance staff and key stakeholders find the system acceptable?	Proportion of surveillance staff that were satisfied with reports and follow-ups	Of the relevant staff interviewed, 17/18, 42/50 and 49/50 reported being satisfied with the virological reports, quarterly bulletins and telephone follow-ups, respectively	3
	Proportion of work time devoted to surveillance activities	37% and 25% for the 50 respondents from the sentinel sites and 17 respondents from the IPM, respectively	2
	Mean annual cost of the surveillance system, for ILI surveillance	US$ 94 364	2
**Flexibility**			
Could the system be easily adapted to cover illnesses other than influenza?	Number of syndromes surveyed under the fever surveillance system	Four: arboviruses, diarrhoea, influenza and malaria	3
	Number of pathogens surveyed under the ILI component of the fever surveillance system	The system can detect up to 14 respiratory viruses	3
**Stability**			
Does the system function smoothly and does it appear sustainable?	Proportion of evaluated weeks during which all sentinel sites were functional	93.3% (291/312)	3
	Proportion of data queries successfully resolved	93.2% (137/147)	3
	Availability and use of SOP for surveillance	Of 50 respondents, 29, 46 and 44 reported making regular use of sample collection, decision tree and surveillance procedures, respectively	3
	Frequency of interruptions in supplies	Of 50 respondents, 28, 9 and 36 reported no interruptions in the supplies of CRF, sampling materials and telephone credit for SMS, respectively	2
	Proportion of sentinel sites with at least one member of staff trained in sentinel surveillance procedures	71.9% (23/32)	2
	Proportion of surveillance staff trained in sentinel surveillance procedures	Training had been received by 66.7% (18/27) of respondents with primary responsibility for surveillance activities and 34.8% (8/23) of respondents who were supporting staff	1
**Utility**			
Does the system provide information that is useful for public health authorities and communities?	Number of ILI alerts detected	In 2014, 38 alerts were detected in 16 sentinel sites	3
	Proportion of sentinel sites – other than those that collected samples routinely – that initiated collection of samples after local ILI alert	72.7% (8/11)	2
	Isolation and sharing of circulating seasonal influenza strains	NIC shared circulating isolates with WHO Collaborating Centres 11 times – out of the 12 requested by WHO	3
	Identification capacity for emerging influenza strains with pandemic potential	NIC successfully passed nine external quality assessments, with an overall score of 98.9%	3
	Proportions of surveillance staff that receive the virological reports, the influenza surveillance reports and influenza bulletins	12 (66.7%) of 18 respondents working at biological sites, 35 (70.0%) of all 50 respondents and 27 (54.0%) of all 50 respondents had reportedly received the virological reports, influenza surveillance reports and the influenza bulletins, respectively	2

CRF: case report forms; ILI: influenza-like illness; IPM: Institut Pasteur de Madagascar; IQR: interquartile range; NIC: national influenza centre; SMS: short message service; SOP: standard operating procedures; US$: United States dollars; WHO: World Health Organization.

^a^ Each quantitative indicator with a percentage value of < 60%, 60–79% and ≥ 80% were given scores of 1, 2 and 3, respectively – representing weak, moderate and good performance, respectively. The qualitative indicators were also scored 1, 2 or 3 but these scores were based on the consensus opinions of 10 surveillance experts – i.e. three virologists, three public health specialists, two epidemiologists and two surveillance officers – who worked at the Institut Pasteur de Madagascar or the Malagasy Ministry of Public Health.

^b^ The key variables evaluated for SMS data were code of sentinel site, date of patient visit and numbers of fever cases, ILI cases and patients. Those evaluated for CRF were absence/presence of fever with cough and/or sore throat, code of sentinel site and dates of patient visit and symptom onset.

^c^ Any of the following were considered to be failures in meeting the sampling criteria: specimen collection tube vial left open; sample without patient identification; no corresponding CRF; no identification of the patient; time between date of onset and date of sampling either ≥ 7 days or not available; time between date of sampling and sample receipt at IPM either ≥ 7 days or not available; sampling kit used after its stated expiry date; no diagnosis of influenza.

Data quality, stability and timeliness were evaluated using the central database at the Institut Pasteur de Madagascar. To evaluate the other five attributes, semi-structured interviews and standardized self-administered questionnaires were used to collect relevant data from 85 individuals involved in the surveillance system from the sentinel sites (68 individuals) and from the Institut Pasteur de Madagascar or the Malagasy Ministry of Public Health (17 individuals). However, 18 staff members from sentinel sites failed to respond.

## Relevant changes

Between January 2009 and December 2014, 177 718 fever cases were reported from the 34 sentinel sites. Overall, 25 809 (14.5%) of these fever cases were considered to have ILI. Samples were collected from 35.6% (9192) of the ILI cases and tested for influenza; 3573 (38.9%) of those tested were found positive. Table 1 summarizes the results of our evaluation of the influenza surveillance component of the fever surveillance system. The data collected on ILI appeared to be of good quality. Full data on most of the cases observed at the sentinel sites were sent in a timely manner. The case definition of ILI and the sampling criteria also appeared to be respected. However, less than 50% (4265/9293) of the samples collected reached the laboratory within 48 hours of their collection. In terms of representativeness, it seems likely that the low median age of the ILI cases observed at the sentinel sites – i.e. four years – reflects a reluctance of adolescents and adults with fever to seek care. More than 80% (47/50) of the staff interviewed stated that the implementation of their surveillance activities was easy and that the time they devoted to such activities was acceptable. Although none of the interviewees reported delays in the collection of samples from patients, 36 (54%) reported regular delays in the collection of case report forms by the express couriers. Over our study period, the mean annual costs of the entire fever surveillance system and the laboratory testing of samples were estimated to be 94 364 and 44 588 United States dollars, respectively.

The fever surveillance system appeared capable of monitoring trends in several fever-associated illnesses under a unified platform and appeared to be quite stable, at least in terms of reporting frequency. Each year the national influenza centre shared the isolates of influenza virus that it had recovered with the WHO Collaborating Centre for Reference and Research on Influenza, London, United Kingdom of Great Britain and Northern Ireland.

## Lessons learnt

The influenza surveillance system showed good performance in terms of most of the indicators and attributes that we evaluated. One of the system’s main strengths was its data quality – including the respect shown to case definition and sampling criteria. The use of mobile phones and texting for the transmission of daily aggregated data, the follow-up and the relative simplicity of the system contributed to improving the completeness, quality and timeliness of the data and the acceptability of the system to sentinel site staff. The main weaknesses that we observed were the frequent shortages of blank case report forms and inadequacies in the number of staff trained. Although half of the surveillance staff interviewed reported that the associated workload was the main challenge in the implementation of surveillance activities, all of them reportedly felt that – given the probable benefits to public health – the time they spent on such activities remained reasonable. The delays between the collection of samples and their receipt in the virological laboratory were another issue.

During our evaluation, we used scores based on an arbitrary scale to estimate the quality of the surveillance system in terms of each of the indicators we evaluated. We decided not to give an overall score for each of the eight attributes we evaluated because the indicators for each attribute are unlikely to have equal importance.

Although the annual costs of the system appeared moderate, the system, at the time of writing, remains entirely supported by external funding. To improve the system’s sustainability, advocacy is needed to promote financial support from the Malagasy Ministry of Health and other national stakeholders. Ideally, the influenza surveillance system should be nested within an integrated system of disease surveillance based on a syndromic approach. If such a system can be kept simple, its acceptability to surveillance staff and its data quality and timeliness are more likely to be good (Box 1). If such a system is going to be sustainable in the long term, the number of sentinel sites and the tests used need to be tailored to the funds available.

Box 1 **Summary of main lessons learnt**

- During 2009–2014, the influenza surveillance system in Madagascar appears to have performed well.
- The system apparently provided reliable and timely data.
- Given its flexibility and overall moderate cost, the system may become a useful model for syndromic and laboratory-based surveillance in other low-resource settings.

Given its flexibility and moderate costs, Madagascar’s influenza surveillance system may be a useful model for syndromic and laboratory-based surveillance in other resource-constrained settings.

## References

[1] Global epidemiological surveillance standards for influenza. Geneva: World Health Organization; 2014. Available from: www.who.int/influenza/resources/documents/influenza_surveillance_manual/en [cited 2016 Nov 31].

[2] Radin JM, Katz MA, Tempia S, Talla Nzussouo N, Davis R, Duque J, et al. Influenza surveillance in 15 countries in Africa, 2006–2010. J Infect Dis. 2012 12 15;206 Suppl 1:S14–21. 10.1093/infdis/jis606 23169960

[3] Rajatonirina S, Heraud JM, Randrianasolo L, Orelle A, Razanajatovo NH, Raoelina YN, et al. Short message service sentinel surveillance of influenza-like illness in Madagascar, 2008–2012. Bull World Health Organ. 2012 5 01;90(5):385–9. 10.2471/BLT.11.097816 22589573 PMC3341687

[4] International Health Regulations (2005) [Internet]. Geneva: World Health Organization; 2008. Available from: http://www.who.int/ihr/publications/9789241596664/en/ [cited 2017 Jan 27].

[5] Randrianasolo L, Raoelina Y, Ratsitorahina M, Ravolomanana L, Andriamandimby S, Heraud JM, et al. Sentinel surveillance system for early outbreak detection in Madagascar. BMC Public Health. 2010 1 21;10(1):31. 10.1186/1471-2458-10-31 20092624 PMC2823701

[6] WHO recommended surveillance standards. Second edition [WHO/CDS/CSR/ISR/99.2]. Geneva: World Health Organization; 1999. Available from: http://www.who.int/csr/resources/publications/surveillance/whocdscsrisr992.pdf [cited 2016 Jun 8].

[7] WHO global epidemiological surveillance standards for influenza. Geneva: World Health Organization; 2012. Available from: http://www.who.int/influenza/resources/documents/influenza_surveillance_manual/en/ [cited 2016 Jun 8].

[8] Razanajatovo NH, Richard V, Hoffmann J, Reynes JM, Razafitrimo GM, Randremanana RV, et al. Viral etiology of influenza-like illnesses in Antananarivo, Madagascar, July 2008 to June 2009. PLoS ONE. 2011 3 03;6(3):e17579. 10.1371/journal.pone.0017579 21390235 PMC3048401

[9] Rajatonirina S, Heraud JM, Orelle A, Randrianasolo L, Razanajatovo N, Rajaona YR, et al. The spread of influenza A(H1N1)pdm09 virus in Madagascar described by a sentinel surveillance network. PLoS ONE. 2012;7(5):e37067. 10.1371/journal.pone.0037067 22615893 PMC3353907

[10] Centers for Disease Control and Prevention (CDC). Guidelines for evaluating surveillance systems. MMWR Suppl. 1988 5 6;37(5):1–18. 3131659

[11] German RR, Lee LM, Horan JM, Milstein RL, Pertowski CA, Waller MN; Guidelines Working Group Centers for Disease Control and Prevention (CDC). Updated guidelines for evaluating public health surveillance systems: recommendations from the Guidelines Working Group. MMWR Recomm Rep. 2001 7 27;50 RR-13:1–35, quiz CE1–7. 18634202

